# Avalanche Multiplication in Two-Dimensional Layered Materials: Principles and Applications

**DOI:** 10.3390/nano15090636

**Published:** 2025-04-22

**Authors:** Zhangxinyu Zhou, Mengyang Kang, Yueyue Fang, Piotr Martyniuk, Hailu Wang

**Affiliations:** 1 State Key Laboratory of Infrared Physics, Shanghai Institute of Technical Physics, Chinese Academy of Sciences, Shanghai 200083, China; zzxykeyan@whut.edu.cn (Z.Z.); 23111110097@stu.xidian.edu.cn (M.K.); fangyueyue22@mails.ucas.ac.cn (Y.F.); piotr.martyniuk@wat.edu.pl (P.M.); 2 School of Material Science and Engineering, Wuhan University of Technology, Wuhan 430070, China; 3 State Key Discipline Laboratory of Wide Band Gap Semiconductor Technology, School of Microelectronics, Xidian University, 2 South Taibai Road, Xi’an 710071, China; 4 University of Chinese Academy of Sciences, Beijing 100049, China; 5 Institute of Applied Physics, Military University of Technology, 2 Kaliskiego St., 00-908 Warsaw, Poland

**Keywords:** two-dimensional materials, avalanche multiplication effect, avalanche photodiodes, impact ionization field-effect transistors, neuromorphic devices

## Abstract

The avalanche multiplication effect, capable of significantly amplifying weak optical or electrical signals, plays a pivotal role in enhancing the performance of electronic and optoelectronic devices. This effect has been widely employed in devices such as avalanche photodiodes, impact ionization avalanche transit time diode, and impact ionization field-effect transistors, enabling diverse applications in biomedical imaging, 3D LIDAR, high-frequency microwave circuits, and optical fiber communications. However, the evolving demands in these fields require avalanche devices with superior performance, including lower power consumption, reduced avalanche threshold energy, higher efficiency, and improved sensitivity. Over the years, significant efforts have been directed towards exploring novel device architectures and multiplication mechanisms. The emergence of two-dimensional (2D) materials, characterized by their exceptional light-matter interaction, tunable bandgaps, and ease of forming junctions, has opened up new avenues for developing high-performance avalanche devices. This review provides an overview of carrier multiplication mechanisms and key performance metrics for avalanche devices. We discuss several device structures leveraging the avalanche multiplication effect, along with their electrical and optoelectronic properties. Furthermore, we highlight representative applications of avalanche devices in logic circuits, optoelectronic components, and neuromorphic computing systems. By synthesizing the principles and applications of the avalanche multiplication effect, this review aims to offer insightful perspectives on future research directions for 2D material-based avalanche devices.

## 1. Introduction

The avalanche multiplication effect refers to a carrier multiplication process where high-energy electrons or holes initiate impact ionization, generating numerous secondary electron-hole pairs. This cascade effect produces substantial signal amplification of weak optical or electrical inputs [1,2,3], making it particularly valuable for enhancing the performance in electronic and optoelectronic devices including avalanche photodiodes (APDs) and impact ionization field-effect transistors (IIFETs). With ongoing technological advancements, avalanche devices based on traditional semiconductor materials, including silicon [4], germanium [5], InGaAs [6,7], HgCdTe [8], and type-II superlattices [9], have developed into a comprehensive technological system.

In an avalanche device, the minimum threshold energy required for avalanche breakdown is approximately equal to the semiconductor’s bandgap energy (*E*_thre_ ≈ *E*_g_) [10,11,12]. However, in conventional semiconductor materials, the carrier multiplication process exhibits low efficiency and requires significantly higher threshold energies—typically 22 times greater than the bandgap energy. This inefficiency arises from two primary factors: the limited density of final states due to momentum conservation constraints and the rapid energy dissipation of carriers through phonon scattering. Consequently, conventional bulk-material avalanche devices require high external bias voltages (typically >100 V for Si and >40 V for InGaAs devices) to initiate impact ionization and sustain avalanche multiplication [13,14,15]. While these devices achieve excellent electrical and optoelectronic performance, the high operating voltages lead to three fundamental limitations: (1) elevated power consumption, (2) increased device noise that degrades the signal-to-noise ratio (SNR), and (3) the need for expensive high-voltage readout circuitry. These factors collectively constrain their applications in low-power electronics and high-SNR optoelectronics [16,17,18]. In response, researchers have begun exploring novel device structures and multiplication mechanisms to overcome these limitations.

In recent years, breakthroughs in large-scale material growth and integration technologies have enabled significant advances in high-performance electronics and optoelectronics [19,20,21]. Among the emerging materials, two-dimensional (2D) materials have garnered considerable attention due to their exceptional mechanical flexibility, strong light-matter interaction, and layer-dependent band structures [22,23,24,25,26,27]. Unlike bulk materials, 2D materials exhibit unique quantum confinement effects that relax the strict momentum conservation requirements, which can significantly influence carrier thermalization processes [28,29,30]. This modified thermalization behavior directly competes with the carrier multiplication mechanism, potentially altering the overall avalanche characteristics [31,32]. This unique property enables carriers to gain sufficient energy for avalanche multiplication at significantly lower voltages, thereby drastically reducing power consumption while simultaneously enhancing sensitivity and noise performance [33,34,35].

In this review, we begin by examining the carrier multiplication mechanism and key performance metrics, followed by a detailed demonstration of typical device structures that leverage the avalanche multiplication effect, along with their corresponding electrical and optoelectronic properties. Additionally, we highlight representative applications of the avalanche multiplication effect, including high-sensitivity photodetection, complementary logic inverters, low-power transistors, and neuromorphic computing devices. Through a systematic integration of fundamental mechanisms and practical implementations, this review aims to offer fresh perspectives to propel the development of next-generation 2D avalanche devices.

## 2. Mechanism and Properties of Avalanche Multiplication Effect

**Figure 1:**
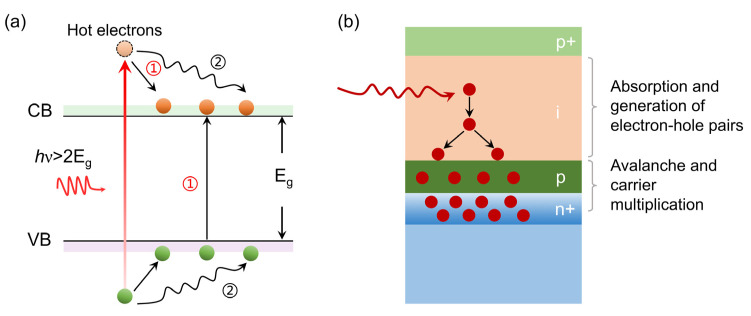
Avalanche multiplication mechanism. (**a**) Carrier multiplication processes in semiconductor devices. Processes ① and ② correspond to carrier multiplication and hot carrier cooling through phonon emission, respectively. (**b**) Impact ionization and multiplication process in avalanche diode structures.

The avalanche multiplication effect of charge carriers is the core working principle of avalanche devices, as illustrated in Figure 1a. In optoelectronic devices, when the photon energy exceeds the bandgap energy of the semiconductor material, the photon energy can be absorbed by electrons, transitioning them to the excited state energy level of the conduction band. At this stage, the electron temperature exceeds the lattice, earning them the designation of “hot electrons”. These hot electrons rapidly release their excess energy and go back to the bottom of the conduction band through phonon scattering processes. If the excess energy surpasses the material’s bandgap, electrons in the valence band can be excited into the conduction band by energy exchange with the hot electrons, thereby achieving carrier multiplication [36,37,38].

When a strong external bias electric field is applied to the semiconductor material, carriers gain sufficient energy to generate additional electron-hole pairs via continuous impact ionization. The newly generated charge carriers are also accelerated by the electric field, leading to the continuous production of more charge carriers and the manifestation of the avalanche effect. This process facilitates carrier multiplication through impact ionization, whereby an initial few charge carriers generate a substantial cascade, dramatically enhancing current flow. As a result, avalanche devices can detect weak signals that are beyond the reach of traditional photodetectors, providing high sensitivity to low-intensity light signals. This capability makes avalanche devices particularly valuable for applications requiring the detection of faint optical signals [39,40].

The multiplication factor or avalanche gain (*M*) is one of the important indicators of avalanche devices, which can reflect the device’s ability to amplify the photocurrent. It is defined as the ratio of the final output photogenerated current to the photogenerated current before multiplication and can be expressed as [41]:

(1) $$M=\frac{I_{p}-I_{d}}{I_{p0}-I_{d0}}$$

where *I_p_* and *I_d_* are the light/dark currents after multiplication, and *I_p_*_0_ and *I_d_*_0_ are the light/dark currents before multiplication. The higher *M* is, the better the detector’s ability to amplify the optical signal. 

Excess noise arises from the stochastic nature of carrier impact ionization during the avalanche multiplication process, presenting significant challenges for the development of accurate theoretical models. Recently, the Hu-Xie avalanche theory was introduced, utilizing a first-principles approach to establish a parameter-free analytical model [42]. This model fundamentally explains the anomalous noise suppression in HgCdTe based on quantum mechanics, playing a pivotal role in advancing avalanche devices. As emphasized by Markus in his recent review, the Hu-Xie theory represents a groundbreaking paradigm for device optimization and noise suppression [43]. The excess noise factor *F*(*M*) is used to characterize the gain instability, which can be expressed as [44]:

(2) $$F\left(M\right)=\frac{\langle M^{2}\rangle }{{\langle M\rangle }^{2}}$$

Based on the local collisional ionization model proposed by McIntyre, the ionization coefficients *α* and *β* for electrons and holes are solely dependent on the electric field. These coefficients are defined as the number of times carriers undergo collisional ionization per unit distance. Specifically, the ratio *k* of the ionization coefficients is given by:

(3) $$k=\frac{\alpha }{\beta }$$

Since *F*(*M*) is only related to *k* for a certain gain, it can be expressed as:

(4) $$F\left(M\right)=kM+\left(1-k\right)\left(2-\frac{1}{M}\right)$$

When *M* is determined, *F*(*M*) decreases as *k* decreases. This provides a theoretical basis for the structural design and material selection of low-noise avalanche devices by increasing (or suppressing) the collision ionization of a carrier, resulting in a decrease in k value and thus reducing *F*(*M*). Due to the constraints imposed by material properties (*α* and *β*) on the ionization coefficient ratio (*k*), when both electrons and holes undergo impact ionization (*k* → 1), charge carriers separate under the electric field, leading to bidirectional carrier transport and chain-reaction avalanche multiplication. In contrast, when *k* → 0, only electrons (or holes) contribute significantly to the multiplication process, enabling comparable gain within a shorter timescale due to unipolar carrier transport. Therefore, by strategically selecting materials that favor single-carrier multiplication (either electrons or holes), avalanche devices can achieve deterministic gain with minimal excess noise.

## 3. Typical Device Structures Based on the Avalanche Multiplication Effect

Traditional material-based avalanche devices exhibit fundamental limitations including elevated power requirements and significant noise generation. In contrast, 2D materials offer an attractive alternative platform due to their monolayer thickness and tunable electronic characteristics. The strong quantum size effect resulting from this nanoscale thickness significantly enhances Coulomb interactions, which greatly facilitates energy exchange between charge carriers, thereby enabling efficient avalanche multiplication [45,46,47]. Additionally, 2D materials exhibit higher exciton binding energy and lower electron-phonon coupling efficiency compared to bulk materials, leading to longer carrier lifetimes [48]. These unique physical properties allow 2D material-based avalanche devices to trigger avalanche multiplication effects at significantly lower bias voltages, paving the way for the development of high-gain, low-noise avalanche devices. Therefore, extensive research has been conducted in this field. Table 1 summarizes the performance parameters of avalanche detectors based on typical bulk materials and 2D materials. The compared metrics include responsivity (*R*), response time (*RT*), operating wavelength (*λ*), dark current (*I*_Dark_), multiplication factor (*M*), operating temperature (*T*), threshold voltage, and external quantum efficiency (*EQE*). While conventional bulk-material avalanche devices exhibit robust performance at room temperature, they inherently require high breakdown voltages (exceeding 30 V). In comparison, two-dimensional material-based devices achieve lower operational voltages due to their small size but often operate with slow response time and low responsivity. Below, we provide a detailed classification and analysis of these structures to better understand their design principles and performance characteristics.

### 3.1. Schottky Junction

**Figure 2:**
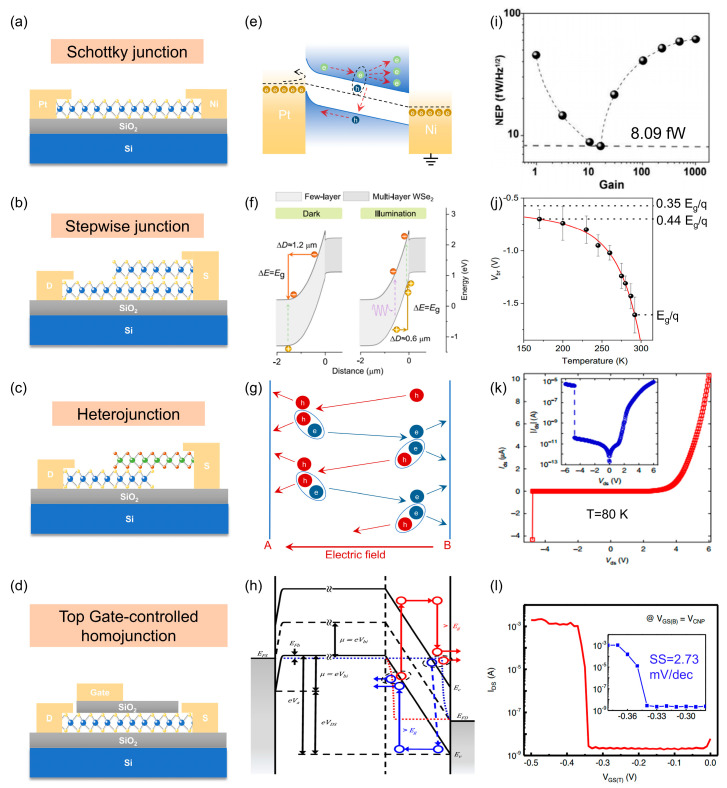
Working principles and performance characteristics of avalanche devices with different structures. (**a**–**d**) Structural models of Schottky junction, stepwise junction, heterojunction, and top gate-controlled homojunction, respectively. Schottky junctions typically employ metals with large work function contrasts, e.g., Pt (~5.65 eV) and Ni (~4.6 eV). (**e**–**h**) Working principles of Pt/Wse_2_/Ni Schottky junction, WSe_2_ stepwise junction, InSe/BP heterojunction, and WSe_2_ top gate-controlled homojunction, respectively. (**i**) NEP curves of Pt/WSe_2_/Ni Schottky junction. (**j**) Reverse breakdown voltage versus operating temperature curve of WSe_2_ stepwise junction. (**k**) *I*_DS_-*V*_DS_ curves of InSe/BP heterojunction. (**l**) Enlarged view of the *I*_DS_-*V*_GS_ characteristics and subthreshold region of the WSe_2_ top gate-controlled homojunction at room temperature.

When metals come into contact with semiconductor materials, potential barriers are typically formed at the interface due to differences in their work functions (Figure 2a) [49,50]. These barriers can effectively block dark charge carriers during avalanche processes, enabling the realization of avalanche devices with high gain and reduced noise [51]. Inspired by this concept, X. Li et al. demonstrated the use of work function matching in WSe_2_ devices to address the inherent gain-noise issue in the process of avalanche multiplication [52]. By strategically designing Schottky barriers, they achieved a significant reduction in noise while maintaining high gain, showcasing a promising approach for optimizing the performance of 2D material-based avalanche devices. As illustrated in Figure 2e, when a high reverse bias is applied, the photogenerated carriers are sufficiently accelerated to initiate the avalanche multiplication effect at a lower electric field, while dark carriers are blocked by the Schottky barriers at the platinum contact. This approach achieves a remarkable gain of 5 × 10^5^, while the noise equivalent power is reduced to 8.09 fW/Hz^1/2^, as shown in Figure 2i, thereby offering a novel perspective for detecting weak signals. Similarly, J. Jia et al. demonstrated avalanche carrier multiplication through the Schottky barrier formed by the Ti/black phosphorus (BP) contact [53]. Their findings indicate that the 2D BP device achieves an external quantum efficiency (EQE) of 310 and a responsivity of 130 A/W, while attaining a signal-to-noise ratio of 100 dB. Additionally, Z. Zhang et al. reported comparable results using a Schottky barrier formed by graphite/InSe with low threshold voltage, but unlike conventional avalanche devices, it exhibits a negative temperature coefficient [54].

### 3.2. Stepwise Junction

J. Kim et al. identified that phonons in transition metal dichalcogenides (TMDs) can be categorized into two modes: in-plane and out-of-plane [39], through testing the carrier multiplication efficiency in thinned WSe_2_ and MoTe_2_ materials. Generally, the out-of-plane mode is more active in electron-phonon (e-p) interactions than the in-plane mode, and the contribution of the out-of-plane mode to e-p scattering can be significantly diminished by reducing the material thickness. According to this discovery, by thinning a portion of a 2D material and shaping it into a stepwise structure with few/multiple layers acting as wide/narrow gap semiconductors, the avalanche process of charge carriers can be triggered earlier. H. Wang et al. utilized mechanical exfoliation technology to fabricate WSe_2_ into the structure illustrated in Figure 2b [55]. In this structure, the pristine homojunction interface eliminates detrimental scattering induced by interface defect states. Meanwhile, the stepwise morphology induces strong localized electric fields that enhance Coulomb interactions between charge carriers while suppressing out-of-plane phonon-mode-dominated scattering processes. This enables the achievement of a threshold energy approaching the theoretical limit *E*_g_ (where *E*_g_ is the semiconductor bandgap) at room temperature. The device exhibits an extremely low dark current (10–100 fA) and achieves detection sensitivity up to 10,000-photon level in the near-infrared range. Recently, the same group further developed an IIFET based on a stepwise van der Waals WSe₂ homojunction. The fabricated device demonstrates a low subthreshold swing (SS) of 3.09 mV/dec and a high multiplication factor exceeding 10^4^ at room temperature [56]. These works provides new perspectives for developing next-generation avalanche devices with high sensitivity, low power consumption, and low threshold voltage.

### 3.3. Heterojunction

When two distinct semiconductors are brought into contact, the application of a relatively small bias voltage can induce significant band bending at the material interface. This band bending enables photogenerated carriers to acquire sufficient energy to trigger avalanche gain at a much lower bias voltage compared to traditional semiconductors [57,58,59]. To achieve a device characterized by high gain, low bias, and low noise, A. Gao et al. constructed a vertical InSe/BP heterojunction structure that leverages the ballistic avalanche phenomenon [60]. This design results in a detector with a low avalanche threshold voltage (approximately 4.8 V) and outstanding noise characteristics. As illustrated in Figure 2g, conventional avalanche devices rely on a cascade process for rapid current multiplication, which generates significant excess noise due to the random generation of carriers. In contrast, the ballistic avalanche process occurs within a nanoscale transport channel, where a hole is energized sufficiently by the electric field to create an electron-hole pair in Region A. Simultaneously, two holes are collected, and electrons are injected back into the channel, initiating a shock ionization process that generates another electron-hole pair in Region B. At this stage, two electrons are collected, and the hole triggers the avalanche process anew. The symmetric energy band structure of BP ensures that the ionization probabilities for electrons and holes are equal, facilitating substantial carrier multiplication without inducing excess noise during the ballistic avalanche process, as no scattering events occur.

For WSe_2_/MoS_2_ two-photon absorbing heterojunctions, B. Son et al. achieved avalanche multiplication of electrons and holes in WSe_2_ and MoS_2_ monolayers [57], respectively, by applying a moderate bias of >6.5 V. The ultrahigh gain of approximately 1300 enhanced the device’s responsivity by nearly three orders of magnitude, reaching 88 μA/W. This work paves the way for the development of photonic integrated circuits and presents new possibilities for the construction of practical infrared detectors.

### 3.4. Top Gate-Controlled Homojunction

To achieve the goals of low power consumption and high energy efficiency, reducing the subthreshold swing has emerged as a critical research focus [61,62,63,64]. This effort has spurred extensive investigations, as summarized in Table 2, which provides detailed performance metrics including the subthreshold swing, the ratio of on-state current to off-state current (on/off ratio), on-state current density (*I*_on_), and off-state current density (*I*_off_). Among these efforts, leveraging the impact ionization mechanism to harness the high-gain characteristics of avalanche breakdown has emerged as an effective strategy for minimizing energy consumption in device operation [65,66]. By employing top-gate modulation on homogeneous WSe_2_ transverse junctions, H. Choi et al. demonstrated exceptional performance, achieving an average SS as low as 2.73 mV/dec and reducing the device’s threshold voltage to below 1 V [67]. Specifically, in the region covered by the top gate, the doping level increases significantly with the application of top-gate voltage, eventually leading to the metallization of this area and effectively shortening the channel length. Simultaneously, in the region without top-gate coverage, the electric field strength is substantially enhanced, enabling carriers to gain sufficient energy to generate electron-hole pairs through impact ionization, thereby facilitating carrier multiplication. This mechanism not only significantly improves the device’s switching characteristics but also provides a novel pathway for the design of energy-efficient electronic devices.

**Table 1:** Performance metrics of bulk and two-dimensional material avalanche devices with different structures.

Structures		Device	*R* (A/W)	*RT* (ms)	*λ* (nm)	*I*_Dark_ (A)	*M*	*T* (K)	Threshold Voltage (V)	*EQE* (%)	Ref.
Bulk materials		Si	50–120	0.1–2 × 10^−6^	400–1100	0.1–1 × 10^−9^	20–400	300	150–400	77	[4]
		Ge	2.5–25	5–8 × 10^−7^	800–1650	5–50 × 10^−8^	50–200	/	20–40	55–75	[5]
		InGaAs	/	1–5 × 10^−7^	1100–1700	1–5 × 10^−8^	10–40	/	20–30	60–70	[6]
Two-dimensional materials	Schottkyjunction	WSe_2_	/	0.05	520	10^−14^	5 × 10^5^	300	15	60	[52]
		MoTe_2_-WS_2_-MoTe_2_	6.02	475	400–700	9.3 × 10^−11^	587	295	10.4	1406	[49]
		InSe	11,000	1	405–785	5 × 10^−9^	500	/	1.3	/	[50]
		BP	130	/	500–1100	2 × 10^−6^	7	300	14.7	31,000	[53]
		InSe	/	0.06	400–800	1.3 × 10^−9^	152	/	12	866	[51]
	Stepwise junction	WSe_2_	/	/	520	1 × 10^−15^	470	300	1.6	/	[55]
		WSe_2_	/	/	/	/	10^4^	300	4	/	[56]
	Heteroj-unction	WSe_2_/MoS_2_	8.8 × 10^−5^	/	532–1030	/	1300	300	6.5	/	[57]
		Gr-MoTe_2_-Gr	5	0.03	600–1350	/	/	300	/	40	[58]
		Gr-MoTe_2_-Gr	0.03	6.15 × 10^−3^	550	6 × 10^−8^	/	300	/	/	[59]
		BP/InSe	80	/	4000	/	10^4^–10^5^	10–180	4.8	2480	[60]
	Top Gate-controlled homojunction	WSe_2_	/	/	/	/	106	300	0.88	/	[67]

Note. *R*: responsivity; *RT*: response time; *λ*: wavelength; *I*_Dark_: dark current; *M*: multiplication factor; *T*: temperature; *EQE*: external quantum efficiency; BP: black phosphorus; Gr: graphene.

**Table 2:** Comparison of the key performance metrics in 2D material-based IIFETs.

Device	Subthreshold Swing (mV/dec)	On/Off Ratio	*I_on_*	*I_off_*	Ref.
WSe_2_	2.73	10^6^	2.29 × 10^−3^ A	1 × 10^−9^ A	[67]
MoS_2_	0.7	10^7^	10^−6^ A/μm	10^−13^A/μm	[61]
Gr/InAs	<0.6	10^6^	2.3 × 10^−4^ A/μm	/	[65]
MoS_2_	11	10^6^	10^−6^ A	10^−12^ A	[62]
Gr/BP/InSe	0.4	>10^5^	1× 10^−6^ A/μm	1.2 × 10^−11^A/μm	[66]
MoS_2_	2.5	10^6^	5.4 × 10^−6^ A/μm	1 × 10^−12^A/μm	[63]
InSe/BP	<0.25	10^4^	1 × 10^−6^ A	1.64 × 10^−10^ A	[60]
MoS_2_	2.26	10^6^	3.9 × 10^−6^ A	1.9 × 10^−12^ A	[64]
WSe_2_	3.09	>10^5^	1 × 10^−6^ A/µm	/	[56]

## 4. Application of Avalanche Multiplication

The avalanche effect, leveraging its unique carrier multiplication mechanism, plays a pivotal role in advancing multiple frontier technologies. In optoelectronic detection, APDs provide substantial signal amplification, enabling high-sensitivity detection of weak optical signals. Silicon-based APDs, utilizing wide-bandgap semiconductor materials, achieve efficient visible-light detection, while InGaAs/InP heterostructure devices—with a spectral response spanning 0.7–1.6 μm—serve as the cornerstone for extending detection capabilities in long-haul fiber-optic communications [68,69]. Beyond telecommunications, the avalanche effect is instrumental in LiDAR systems, where it facilitates the detection of low-reflectivity objects and enables high-resolution 3D imaging for autonomous driving applications. In high-frequency electronics, avalanche transit-time diodes (IMPATT diodes) exploit the interplay between impact ionization and carrier transit-time delay to generate millimeter-wave and terahertz signals, making them vital for next-generation wireless and sensing technologies [2,70]. The biomedical field also benefits from avalanche-based detectors, such as APDs and single-photon avalanche diode (SPAD) arrays, which enhance fluorescence-based imaging, surgical navigation, and high-resolution tomography [71,72]. However, the growing demands of artificial intelligence—particularly in computational imaging and edge sensing—pose new challenges for avalanche devices in terms of speed, noise performance, and integration density. This section will examine recent progress in avalanche-effect applications across these pioneering fields, with particular emphasis on performance optimization strategies and emerging hybrid architectures.

**Figure 3:**
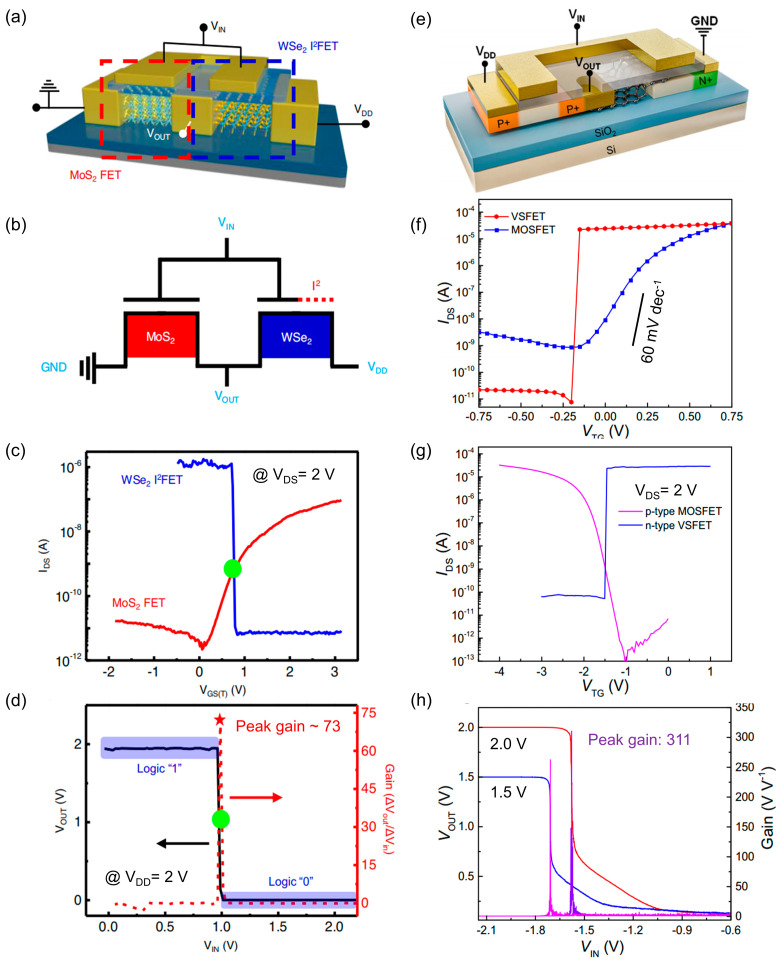
Principles and characteristics of complementary inverter. (**a**,**b**) Structure and schematic diagrams of the complementary inverter based on WSe_2_. (**c**) I_DS_-V_GS_ characteristic curves of WSe_2_ and MoS_2_ devices. (**d**) Operating characteristic curves of the complementary inverter based on WSe_2_ in series with MoS_2_ device. The star indicates that the device achieved a high inverter gain of approximately 73. (**e**) Schematic of the structure of the VSFET-based complementary inverter. (**f**) Transfer characteristic curves of the VSFET versus the MOSFET at V_DS_ = 3 V, where the VSFET exhibits excellent SS. (**g**) Transfer characteristic curves of the VSFET versus the MOSFET during complementary inverter operation. (**h**) Voltage transfer characteristic and gain of the inverter.

As the integration density of transistors on a single chip continues to increase, power consumption has become a critical challenge. To minimize the power consumption of individual transistors, it is essential to achieve the lowest possible SS, enabling rapid switching between the “0” and “1” states. H. Choi et al. demonstrated this principle using a complementary inverter composed of p-type WSe_2_ and n-type MoS_2_ connected in series [67]. This complementary inverter dynamically adjusts the output signal state based on the input signal level. When the input voltage (V_in_) is high, the electron-dominated characteristics of n-type MoS_2_ pull the output voltage (V_out_) low. Conversely, when V_in_ is low, the hole impact ionization in p-type WSe_2_ drives the V_out_ high. The exceptional subthreshold swing of the WSe_2_ device allows the complementary inverter to achieve a low switching threshold voltage, as illustrated in Figure 3c,d, and a high inverter gain of approximately 73. Additionally, this study highlights that adjusting device parameters can yield different threshold voltages, making these devices highly adaptable for practical circuit applications. Similarly, B. Yuan et al. developed vertical layered semiconductor field-effect transistor (VSFET) devices with outstanding subthreshold swing [73], achieving a voltage gain of up to 311 at a 2 V supply voltage by integrating a p-type MOSFET with an n-type VSFET configured as a complementary phase shifter. These advancements underscore the potential of leveraging 2D materials for low-power, high-performance electronic devices.

**Figure 4:**
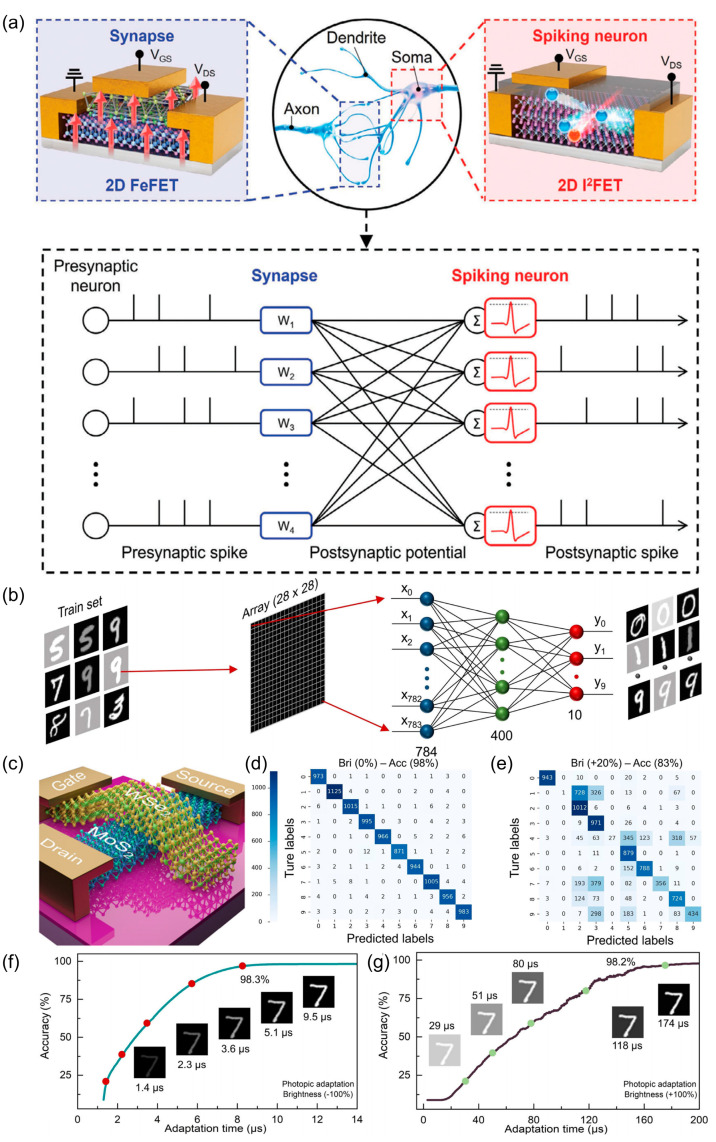
Neuromorphic computing systems. (**a**) Schematic of the peak neural network structure and framework. (**b**) Machine vision image recognition based on convolutional neural networks. (**c**) Diagram of adaptive machine vision device. (**d**) Confusion matrix and its recognition accuracy at standard vs. (**e**) +20% luminance. (**f**,**g**) Recognition rate as a function of time for adaptive machine vision for (**f**) darkness and (**g**) lightness adaptation.

As artificial neural networks continue to advance, their architectures are becoming increasingly complex, leading to higher power consumption. Consequently, avalanche devices with steep SS have attracted considerable attention. By leveraging the high-energy spikes generated by impact ionization during transient time and the ability to control these spikes through operating voltage, these devices can achieve ultra-low power consumption via low operating voltages and fast response times. Furthermore, when integrated with advanced algorithms, they enable energy-efficient neural networks capable of performing complex tasks such as computation and recognition. As shown in Figure 4a, H. Choi et al. employed 2D WSe_2_ shock transistors as synapses in conjunction with 2D ferroelectric transistor neurons to construct a neural network [74], achieving remarkably low energy consumption of 2 pJ per single pulse. Additionally, through the synergistic processing capabilities of these components, an accuracy of 87.5% in face classification was achieved after 20 learning sessions using unsupervised learning. Moreover, the MoS_2_/WSe_2_ heterojunction developed by L. Li et al. demonstrated ultrafast dark and light adaptation processes of 108 μs and 268 μs, respectively, facilitated by the avalanche effect, with switching behavior controlled by the top gate voltage [75]. When combined with deep learning techniques from convolutional neural networks, this system achieved over 98% image recognition accuracy under both dim and bright conditions, as illustrated in Figure 4f,g. These results highlight the significant potential of avalanche photodetectors in the field of bionics, paving the way for energy-efficient and high-performance neuromorphic computing systems.

## 5. Conclusions

Avalanche devices based on traditional semiconductor materials have achieved industrialization and are widely utilized across various fields. However, as technology continues to advance, emerging applications such as low-power artificial neural networks, bionic vision, and low-light detection have imposed higher performance demands on detectors. In this context, avalanche devices based on 2D materials have rapidly evolved, demonstrating exceptional performance in sensitivity, response time, and power consumption. In this review, we provide a concise introduction to the avalanche multiplication mechanism and its key performance metrics, followed by a detailed discussion of the typical structures and applications of 2D material-based avalanche devices. By synthesizing these insights, we aim to offer valuable guidance for the development of more advanced and efficient avalanche devices in the future.
